# Conservation machine learning: a case study of random forests

**DOI:** 10.1038/s41598-021-83247-4

**Published:** 2021-02-11

**Authors:** Moshe Sipper, Jason H. Moore

**Affiliations:** 1 Institute for Biomedical Informatics, University of Pennsylvania, Philadelphia, PA 19104-6021 USA; 2 Department of Computer Science, Ben-Gurion University, Beer Sheva, 84105 Israel

**Keywords:** Computational science, Computer science, Scientific data

## Abstract

Conservation machine learning conserves models across runs, users, and experiments—and puts them to good use. We have previously shown the merit of this idea through a small-scale preliminary experiment, involving a single dataset source, 10 datasets, and a single so-called cultivation method—used to produce the final ensemble. In this paper, focusing on classification tasks, we perform extensive experimentation with conservation random forests, involving 5 cultivation methods (including a novel one introduced herein—*lexigarden*), 6 dataset sources, and 31 datasets. We show that significant improvement can be attained by making use of models we are already in possession of anyway, and envisage the possibility of repositories of *models* (not merely datasets, solutions, or code), which could be made available to everyone, thus having conservation live up to its name, furthering the cause of data and computational science.

## Introduction

We recently presented the idea of *conservation machine learning*, wherein machine learning (ML) models are saved across multiple runs, users, and experiments^1^. Conservation ML is essentially an “add-on” meta-algorithm, which can be applied to any collection of models (or even sub-models), however they were obtained: via ensemble or non-ensemble methods, collected over multiple runs, gathered from different modelers, a priori intended to be used in conjunction with others—or simply plucked a posteriori, and so forth.

A random forest (RF) is an oft-used ensemble technique that employs a forest of decision-tree classifiers on various sub-samples of the dataset, with random subsets of the features for node splits. It uses majority voting (for classification problems) or averaging (for regression problems) to improve predictive accuracy and control over-fitting^2^.

Reference^3^ presented a method for constructing ensembles from libraries of thousands of models. They used a simple hill-climbing procedure to build the final ensemble, and successfully tested their method on 7 problems. They further examined three alternatives to their selection procedure to reduce overfitting. Pooling algorithms, such as stacked generalization^4^, and super learner^5^, have also proven successful. There also exists a body of knowledge regarding ensemble pruning^6^.

We believe the novelty of conservation machine learning, herein applied to random forests, is two-fold. First and foremost, we envisage the possibility of vast repositories of *models* (not merely datasets, solutions, or code). Consider the common case wherein several research groups have been tackling an extremely hard problem (e.g.,^7^), each group running variegated ML algorithms over several months (maybe years). It would not be hard to imagine that the number of models produced over time would run into the millions (quite easily more). Most of these models would be discarded unflinchingly, with only a minute handful retained, and possibly reported upon in the literature. We advocate making all models available to everyone, thus having conservation live up to its name, furthering the cause of data and computational science. Our second contribution in this paper is the introduction of a new ensemble cultivation method—*lexigarden*.

In^1^ we offered a discussion and a preliminary proof-of-concept of conservation ML, involving a single dataset source, 10 datasets, and a single so-called cultivation method. Herein, focusing on classification tasks, we perform extensive experimentation involving 5 cultivation methods, including the newly introduced lexigarden (“Ensemble cultivation”), 6 dataset sources, and 31 datasets (“Datasets”). Upon describing the setup (“Experimental setup”), we show promising results (“Results”), followed by a discussion (“Discussion”) and concluding remarks (“Concluding remarks”).

## Ensemble cultivation

Conservation ML begins with amassing a collection of models—through whatever means. Herein, we will collect models saved over multiple runs of RF training.

Once in possession of a collection of fitted models it is time to produce a final ensemble. We examine five ways of doing so:

1. *Jungle.* Use *all* collected fitted models to form class predictions through majority voting, where each model votes for a single class.
2. *Super-ensemble.* Use an ensemble of ensembles, namely an ensemble of RFs, with prediction done through majority voting, where each RF votes for a single class.
  To clarify, assume we perform 100 runs of RFs of size 100. We are then in possession of a jungle of size 10,000 decision trees, and a super-ensemble of size 100 RFs. Both perform predictions through majority voting.
3. *Order-based (or ranking-based) pruning.* We implemented two methods of ensemble pruning^6^. The first, ranking-based, sorts the jungle from best model (over training set) to worst, and then selects the top *n* models for the ensemble, with *n* being a specified parameter
4. *Clustering-based pruning.* The second ensemble-pruning method performs k-means clustering over all model output vectors, with a given number of clusters, *k*, and then produces an ensemble by collecting the top-scoring (over training set) model of each cluster.
5. *Lexigarden*. A new method, introduce herein and described below, based on lexicase selection, a performant *selection* technique for evolutionary algorithms, with selection being one of the primary steps in such algorithms^8,9^. This step mimics the role of natural selection in nature, by probabilistically selecting fitter individuals from the evolving population, which will then undergo pseudo-genetic modification operators.

Lexicase selection selects individuals by filtering a pool of individuals which, before filtering, typically contains the entire population. The filtering is accomplished in steps, each of which filters according to performance on a single test case. Lexicase selection has been used productively within the field of evolutionary algorithms^10,11^. Herein, we co-opt it to cultivate a “garden” of select trees from the jungle, introducing the *lexigarden* algorithm. Lexigarden generates a garden of a specified size, whose models were selected through lexicase selection.

Algorithm 1 provides the pseudocode. Lexigarden receives a jungle of models, a dataset along with target values, and the number of models the generated garden is to contain. The lexicase function begins by randomly shuffling the dataset, after which it successively iterates through it, retaining only the models that provide a correct answer for each instance. In the end we are left with either a single model or a small number of models, which are precisely those that have correctly classified the subset of the dataset engendered through the looping process. The lexicase function is called as many times as needed to fill the garden with models. Lexigarden ends up generating a highly diverse subset of all the models by picking ones that each excels on a particular random subset of instances.

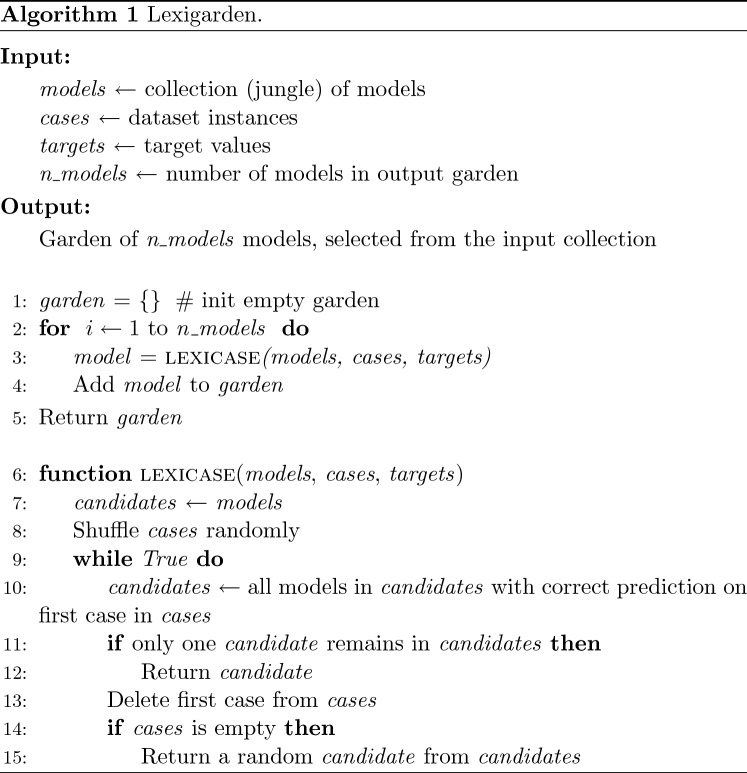



## Datasets

To compose a variegated collection of classification datasets for the experiments described in the next section we turned to 6 different sources:

1. **Easy**. Scikit-learn’s^12^ “easy” classification datasets, where near-perfect performance is expected as par for the course: iris, wine, cancer, digits.
2. **Clf**. Datasets produced through make_classification, a Scikit-learn function that, “initially creates clusters of points normally distributed (std=1) about vertices of an n_informative-dimensional hypercube with sides of length 2*class_sep and assigns an equal number of clusters to each class.”^13^
3. **HIBACHI**. A method and software for simulating complex biological and biomedical data^14^.
4. **GAMETES**. Software that generates complex biallelic single nucleotide polymorphism (SNP) disease models for simulation studies. We generated a 2-way and a 3-way biallelic pure, strict, epistatic model with a heritability of 1, referred to in related work as the Xor model^15^.
5. **OpenML**. A repository of over 21,000 datasets, of which we selected problems designated as “Highest Impact”, with a mix of number of samples, features, and classes^16^.
6. **PMLB**. The Penn Machine Learning Benchmark repository is an accessible, curated, and developing public benchmark resource to facilitate identification of the strengths and weaknesses of different machine learning methodologies^17^.

**Figure 1:**
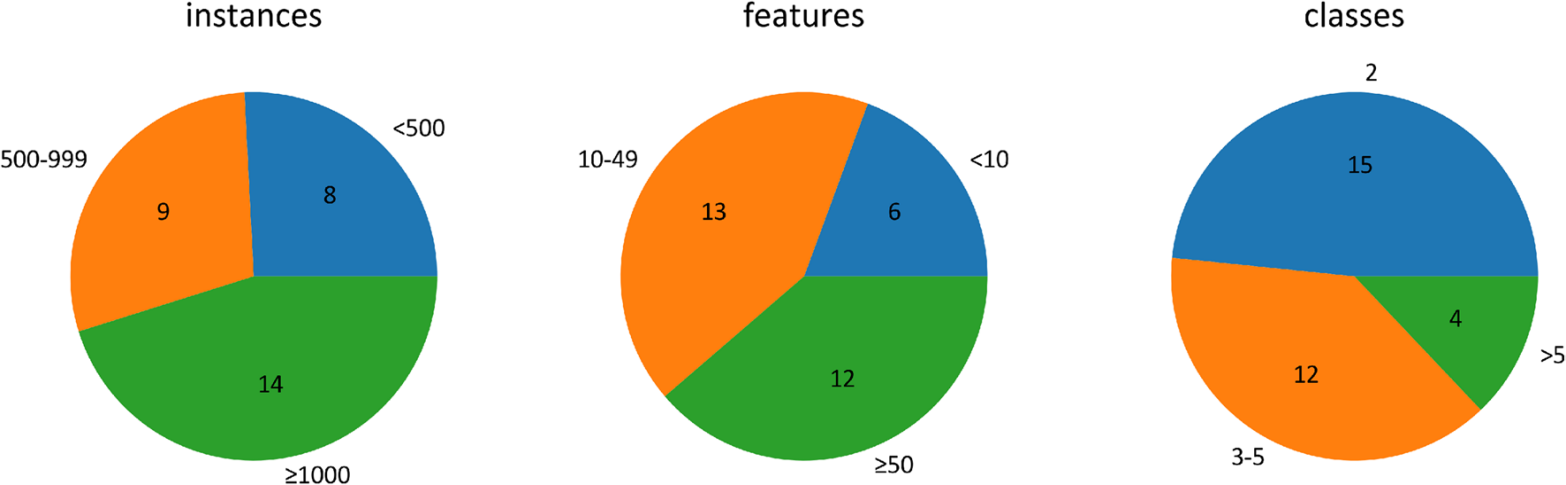
A “bird’s-eye view” of the 31 datasets used in this study: number of instances (left), number of features (center), and number of classes (right).

Note that in addition to there being 6 dataset sources, there is also a mix of dataset repositories (Easy, OpenML, PMLB) and dataset generators (Clf, HIBACHI, GAMETES). Figure 1 shows a “bird’s-eye view” of the total of 31 datasets.

## Experimental setup

The experiments were based on Scikit-learn’s RandomForestClassifier function, which we used with its default values (our aim here was not to improve RFs per se but to show that conservation can significantly improve a base ML algorithm)^12,13^.

The experimental setup is shown in Algorithm 2. For each replicate experiment we created 5 folds, for 5-fold cross validation. For each fold the dataset was split into a training set of 4 folds and the left-out test fold. 100 runs were conducted per fold. Each run consisted of fitting a 100-tree RF to the traning set and testing the fitted RF on the test set. In addition, all trees were saved into a jungle and all RFs were saved into a super-ensemble; these could then be tested on the test set.

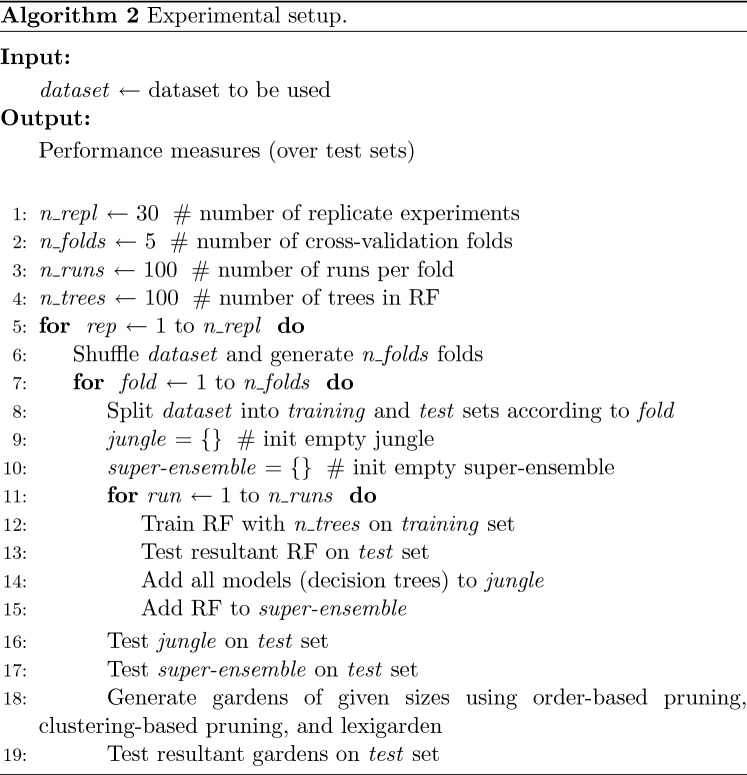



The jungle also served as fodder for the cultivation methods of “Ensemble cultivation”. For every training epoch, we generated gardens of given sizes (see Table 1) using the three cultivation methods: order-based pruning, clustering-based pruning, and lexigarden. These gardens could then also be tested on the test set.

## Results

Table 1 shows the results of our experiments. Each line in the table presents the results for a single dataset, involving 30 replicate experiments, as delineated in “Experimental setup”. We show the mean performance of RFs alongside the improvement of the 5 conservation methods discussed in “Ensemble cultivation”.

**Table 1:** Experimental results.

Dataset	Samp	Feat	Info	Cls	RF	Jung	Sup	Ord300	Ord1000	Clus20	Clus50	Lex300	Lex1000
Easy													
iris	150	4	–	3	0.95 (0.03)	0.2%	0.1%	− 0.1%	− 0.1%	− 0.3%	− 0.1%	− 0.0%	− 0.1%
wine	178	13	–	3	0.98 (0.02)	0.1%	0.1%	− 0.6% (!!)	− 0.2%	− 0.7% (!!)	− 0.2%	− 1.0% (!!)	− 1.0% (!!)
cancer	569	30	–	2	0.96 (0.02)	0.0%	0.1%	0.5% (!!)	0.5% (!)	0.1%	0.4% (!)	0.1%	0.2%
digits	1797	64	–	10	0.98 (0.01)	0.2% (!)	0.2% (!)	0.0%	0.1% (!)	− 0.9% (!!)	− 0.3% (!!)	0.1% (!)	0.2% (!)
Clf													
clf	500	400	200	4	0.33 (0.05)	33.0% (!!)	31.1% (!!)	14.3% (!!)	25.3% (!!)	− 12.1% (!!)	− 4.7% (!!)	13.8% (!!)	22.8% (!!)
clf	500	400	200	10	0.13 (0.04)	48.5% (!!)	45.5% (!!)	14.7% (!!)	34.3% (!!)	− 13.2% (!!)	− 6.0% (!)	19.7% (!!)	37.0% (!!)
clf	1000	100	90	2	0.78 (0.03)	5.5% (!!)	5.2% (!!)	3.1% (!!)	4.2% (!!)	− 8.6% (!!)	− 3.1% (!!)	3.4% (!!)	4.5% (!!)
clf	1000	200	90	3	0.58 (0.03)	20.3% (!!)	20.1% (!!)	12.6% (!!)	17.6% (!!)	− 16.2% (!!)	− 4.7% (!!)	11.3% (!!)	17.1% (!!)
clf	1000	300	200	4	0.40 (0.03)	38.6% (!!)	37.4% (!!)	18.6% (!!)	30.9% (!!)	− 17.7% (!!)	− 7.5% (!!)	16.4% (!!)	29.5% (!!)
clf	1000	500	400	5	0.27 (0.03)	54.0% (!!)	49.6% (!!)	17.7% (!!)	38.0% (!!)	− 13.3% (!!)	− 7.3% (!!)	14.5% (!!)	35.8% (!!)
clf	1000	1000	900	10	0.11 (0.02)	45.2% (!!)	37.4% (!!)	9.1% (!!)	21.9% (!!)	− 7.9% (!!)	− 3.6% (!)	5.7% (!!)	24.8% (!!)
HIBACHI													
hibachi1	999	10	–	2	0.87 (0.03)	2.3% (!!)	2.2% (!!)	10.3% (!!)	10.0% (!!)	− 6.5% (!!)	− 8.2% (!!)	10.4% (!!)	10.4% (!!)
hibachi2	999	10	–	2	0.65 (0.04)	3.2% (!!)	3.0% (!!)	47.5% (!!)	41.1% (!!)	19.7% (!!)	16.7% (!!)	47.5% (!!)	47.7% (!!)
hibachi3	999	10	–	2	0.61 (0.04)	1.8% (!)	1.6% (!)	53.9% (!!)	44.5% (!!)	19.6% (!!)	15.8% (!!)	55.3% (!!)	55.6% (!!)
hibachi4	999	10	–	2	0.83 (0.03)	2.4% (!!)	2.5% (!!)	7.9% (!!)	8.1% (!!)	0.5%	3.2% (!!)	7.1% (!!)	7.5% (!!)
hibachi5	999	10	–	2	0.84 (0.04)	2.9% (!!)	2.7% (!!)	17.1% (!!)	16.4% (!!)	− 6.8% (!!)	− 8.1% (!!)	17.2% (!!)	17.2% (!!)
GAMETES													
xor2	1600	20	2	2	0.91 (0.03)	3.4% (!!)	3.3% (!!)	10.1% (!!)	10.1% (!!)	− 10.9% (!!)	− 13.1% (!!)	9.9% (!!)	9.9% (!!)
xor3	1600	20	3	2	0.57 (0.03)	1.6% (!!)	1.6% (!!)	57.0% (!!)	37.3% (!!)	40.2% (!!)	32.8% (!!)	36.8% (!!)	38.7% (!!)
OpenML													
tA	151	6	–	3	0.71 (0.08)	0.7%	0.8%	13.8% (!!)	8.6% (!!)	17.0% (!!)	14.6% (!!)	17.5% (!!)	18.0% (!!)
arcene	200	10000	–	2	0.81 (0.07)	0.7%	0.8%	2.8% (!!)	2.3% (!!)	2.0% (!)	2.1% (!)	2.8% (!!)	2.9% (!!)
monks	601	6	–	2	0.91 (0.04)	0.8% (!)	0.5%	7.1% (!!)	6.4% (!!)	6.4% (!!)	5.7% (!!)	7.2% (!!)	7.4% (!!)
100	1600	64	–	100	0.83 (0.02)	3.3% (!!)	3.2% (!!)	2.0% (!!)	2.8% (!!)	− 10.1% (!!)	− 2.9% (!!)	2.1% (!!)	2.9% (!!)
madelon	2600	500	–	2	0.70 (0.02)	3.4% (!!)	3.6% (!!)	13.5% (!!)	10.4% (!!)	4.9% (!!)	7.7% (!!)	6.7% (!!)	7.1% (!!)
eye	10936	27	–	3	0.69 (0.01 )	2.2% (!!)	2.2% (!!)	5.9% (!!)	5.2% (!!)	− 1.3% (!!)	1.4% (!!)	2.0% (!!)	2.5% (!!)
PMLB													
cloud	108	7	–	4	0.43 (0.10)	2.4%	2.2%	20.9% (!!)	16.5% (!!)	21.7% (!!)	22.5% (!!)	20.0% (!!)	20.6% (!!)
sonar	208	60	–	2	0.82 (0.06)	1.0%	0.5%	2.2% (!!)	2.1% (!!)	− 0.9%	1.1%	1.6% (!)	2.2% (!!)
biomed	209	8	–	2	0.89 (0.05)	0.0%	0.2%	1.8% (!!)	1.5% (!!)	1.7% (!!)	2.4% (!!)	1.6% (!!)	1.8% (!!)
cars1	392	7	–	3	0.78 (0.06)	0.7%	0.7%	3.1% (!!)	2.5% (!!)	2.4% (!!)	2.7% (!!)	2.8% (!!)	2.3% (!!)
car	1728	21	–	4	0.88 (0.04)	1.2% (!)	0.9% (!)	6.8% (!!)	6.0% (!!)	5.6% (!!)	5.6% (!!)	5.0% (!!)	5.2% (!!)
churn	5000	20	–	2	0.87 (0.02)	0.3% (!)	0.2%	1.7% (!!)	1.5% (!!)	0.2%	0.6% (!!)	1.0% (!!)	1.0% (!!)
allrep	3772	29	–	4	0.53 (0.08)	0.8%	0.3%	31.9% (!!)	26.1% (!!)	25.7% (!!)	25.4% (!!)	27.9% (!!)	28.1% (!!)

Each line shows the results of 30 replicate experiments with 5-fold cross validation. *Dataset*: dataset name. For readability we shortened long dataset names: tA (teachingAssistant), monks (monks-problems-2), 100 (one-hundred-plants-margin), eye (eye_movements), car (car-evaluation). *Samp*: number of dataset samples. *Feat*: number of features. *Info*: number of informative features (when known). *Cls*: number of target classes. *RF*: mean performance of random forests on test set across all replicates (with standard deviation in parentheses). Performance is measured as the balanced accuracy score between target and predicted values. *Jung*: results for jungle of size 10,000 decision trees, comprising percent improvement over RFs. For this and the subsequent improvement results we provide an indication of the p-value of a 10,000-round permutation test of the improvement: a ‘!!’ in parentheses indicates a p-value $< 0.001$, a ‘!’ indicates a p-value $< 0.05$, and no parenthetic value indicates a p-value $>= 0.05$. *Sup*: results for super-ensemble of size 100 RFs. *Ord300*: results for order-based pruning, producing an ensemble of top 300 decision trees. *Ord1000*: results for order-based pruning, producing an ensemble of top 1000 decision trees. *Clus20*: results for clustering-based pruning, with 20 clusters. *Clus50*: results for clustering-based pruning, with 50 clusters. *Lex300*: results for garden of size 300 decision trees, generated by lexigarden. *Lex1000*: results for garden of size 1000 decision trees, generated by lexigarden.

In addition to reporting improvement values we also report their statistical significance, as assessed by 10,000-round permutation tests. The permutation tests focused on the mean test scores across all replicates and folds, comparing each ensemble (mean) to the original RFs. We report on two p-value significance levels: $<0.001$ and $<0.05$.

## Discussion

Focusing on random forests for classification we performed a study of the newly introduced idea of conservation machine learning. It is interesting to note that—case in point—our experiments herein alone produced almost fifty million models (31 datasets $\times$ 30 replicates $\times$ 5 folds $\times$ 100 runs $\times$ 100 decision trees = 46,500,000).

As can be seen in Table 1, we attained statistically significant improvement for all datasets, except for the four “easy” problems, where a standard RF’s accuracy was close to 1 to begin with, and little improvment could be eked out. All but the super-ensemble method ranked highest on several datasets: the jungle method attained the best improvement for 8 datasets, order-based pruning attained the best improvement for 10 datasets, clustering-based pruning attained the best improvement for 2 datasets, and lexigarden attained the best improvement for 8 datasets (we excluded “easy” datasets from this count).

In summary, our results show that conservation random forests are able to improve performance through ensemble cultivation by making use of models we are already in possession of anyway.

There is a cost attached to conserving models, involving memory and computation time. The former is probably less significant, since saving millions and even billions of models requires storage space well within our reach. Computation time of a jungle or super-ensemble is obviously more costly than an ensemble of a lesser size (or a single model), but if the performance benefits are deemed worthwhile then time should not pose an issue. Computing a garden’s output involves only a minor increase in computation time, and a one-time computation of the garden’s members. As pointed out recently by^18^, improvements in software, algorithms, and hardware architecture can bring a much-needed boost. For example, they showed that a sample Python program, when coded in Java, produced a 10.8$\times$ speedup, and coding it in C produced a 47$\times$ speedup; moreover, tailoring the code to exploit specific features of the hardware gained an additional 1300$\times$ speedup.

## Concluding remarks

There are many possible avenues for future exploration:

- We focused on classification tasks, which leaves open the exploration of other types of tasks, such as regression and clustering.
- We examined random forests, with other forms of ensemble techniques—such as sequential, boosting algorithms—yet to be explored.
- Non-ensemble techniques deserve study as well.
- It is also possible to amass models that were obtained through different ML methods (or even non-ML methods).
- We used simple majority voting to compute the output of jungles, super-ensembles, and gardens. More sophisticated methods could be explored.
- We offered lexigarden as a novel supplement to the cultivation-method toolkit. Other cultivation techniques could be devised.
- As noted in^1^, current cloud repositories usually store code, datasets, and leaderboards. One might consider a new kind of model archive, storing a plethora of models, which could provide bountiful grist for the ML mill.

While we focused on random forests herein, we note again that conservation ML is essentially an “add-on” meta-algorithm that can be applied to any collection of models, however they were obtained.

We hope to see this idea receiving attention in the future.

## Data Availability

The code is available at https://github.com/EpistasisLab.
